# A data augmentation approach for a class of statistical inference problems

**DOI:** 10.1371/journal.pone.0208499

**Published:** 2018-12-10

**Authors:** Rodrigo Carvajal, Rafael Orellana, Dimitrios Katselis, Pedro Escárate, Juan Carlos Agüero

**Affiliations:** 1 Electronics Engineering Department, Universidad Técnica Federico Santa María, Valparaíso, Chile; 2 Universidad de Los Andes, Mérida, Venezuela; 3 Coordinated Science Laboratory and Information Trust Institute, University of Illinois, Urbana-Champaign, Illinois, United States of America; 4 Large Binocular Telescope Observatory, Steward Observatory, University of Arizona, Tucson, AZ, United States of America; 5 Instituto de Electricidad y Electrónica, Facultad de Ciencias de la Ingeniería, Universidad Austral, Valdivia, Chile; University of Hong Kong, HONG KONG

## Abstract

We present an algorithm for a class of statistical inference problems. The main idea is to reformulate the inference problem as an optimization procedure, based on the generation of surrogate (auxiliary) functions. This approach is motivated by the MM algorithm, combined with the systematic and iterative structure of the Expectation-Maximization algorithm. The resulting algorithm can deal with hidden variables in Maximum Likelihood and Maximum a Posteriori estimation problems, Instrumental Variables, Regularized Optimization and Constrained Optimization problems. The advantage of the proposed algorithm is to provide a systematic procedure to build surrogate functions for a class of problems where hidden variables are usually involved. Numerical examples show the benefits of the proposed approach.

## 1 Introduction

Problems in statistics and system identification often involve variables for which measurements are not available. Among others, real-life examples can be found in communication systems [1, 2] and systems with quantized data [3, 4]. In Maximum Likelihood (ML) estimation problems, the *likelihood function* is in general difficult to optimize by using closed-form expressions, and numerical approximations are usually cumbersome. These difficulties are traditionally avoided by the utilization of the Expectation-Maximization (EM) algorithm [5], where a surrogate (auxiliary) function is optimized instead of the main objective function. This surrogate function includes the complete data, i.e. the measurements and the random variables for which there are no measurements. The incorporation of such *hidden data* or *latent variables* is usually termed as *data augmentation*, where the main goal is to obtain, in general, simple and fast algorithms [6].

On the other hand, the MM (MM stands for Maximization-Minorization or Minimization-Majorization, depending on the optimization problem that needs to be solved) algorithm [7] is generally employed for solving more general optimization problems, not only for ML and Maximum a Posteriori (MAP) estimation problems. In general, the main motivation for using the MM algorithm is the lack of closed-form expressions for the solution of the optimization problem or dealing with objective cost functions that are not convex. Applications where the MM algorithm has been utilized include communication systems problems [8] and image processing [9]. For constrained optimization problems, an elegant solution is presented by Marks and Wright [10], where the constraints are incorporated via the formulation of surrogate functions. Surprisingly, Marks’ approach has not received the same attention from the scientific community when it comes to compare it with the EM and the MM algorithms. In fact, these three approaches are contemporary, but the EM algorithm has attracted most of the attention (out of the three methods), and it has been used for solving linear and nonlinear statistical inference problems in biology and engineering, see e.g. [11–16], amongst others. On the other hand, as shown in [7], the MM algorithm has obtained much less attention, while Marks’ approach is mostly known to a limited audience in the the Communication Systems community. These three approaches have important similarities: i) a surrogate function is defined and optimized in place of the original optimization problem, and ii) the solution is obtained iteratively. In general, these algorithms are “principles and recipes” [17] or a “philosophy” [7] for constructing solutions to a broad variety of optimization problems.

In this paper we adopt the ideas behind [5, 7, 10] to develop an algorithm for a special class of functions. Our approach generalizes the ones of [5, 7, 10] by reinterpreting the E-step in the EM algorithm and expressing the cost function in terms of an infinite mixture or kernel. This kind of problems can be interpreted as *inverse problems* that, in turn, can be posed as integral equations, such as channel modelling in wireless communications [18], estimation of the distribution of stellar rotational velocities [19], and mass estimation in particle physics problems [20, 21]. In particular cases, the kernel corresponds to a variance-mean Gaussian mixture (VMGM), see e.g. [22]. VMGMs, also referred to as normal variance-mean mixtures [23] and normal scale mixtures [24], have been considered in the literature for formulating EM-based approaches to solve ML [25] and MAP problems, including regularized sparse estimation problems [22, 26, 27]. Sparse estimation problems have been widely studied in the last two decades and several techniques have been developed that include different types of penalties or contraint, see e.g [28, 29] and different strategies for the formulation of those penalties/constraints [30]. In this paper we show that our proposal can also be considered for sparsity problems, however the analysis of the solution is out of the scope of the paper. Our approach is applicable to a wide class of functions, which allows for defining the likelihood function, the prior density function, and constraints as kernels, extending also the work in [10]. Thus, our work encompasses the following contributions: i) a systematic approach to constructing surrogate functions for a class of cost functions and constraints, ii) a class of kernels where the unknown quantities of the algorithm can be easily computed, and iii) a generalization of [5, 7, 10] by including the cost function and the constraints in one general expression. Our proposal is based, among other things, on a particular way to apply Jensen’s inequality [31]. In addition, we provide the details on how to construct quadratic surrogate functions for cost functions and constraints.

Our algorithm is tested by two examples. In the first one we considered the problem of estimating the rotational velocities of stars. The system model corresponds to the convolution of two probability density functions (pdf’s) and thus it is an infinite mixture. We show that our reinterpretation of the EM algorithm allows for the direct application of our proposal for the correct estimation of the parameter of interest. In the second example, we considered the estimation of the channel in wireless communications, where the true distribution can be either Rayleigh or Rice, depending on environment where the electromagnetic waves propagate. The problem is solved considering a sum of a Rayleigh and a Rice term, allowing for a more complex channel distribution. To select the more representative distribution, Akaike’s Information Criterion [32] was also considered in order to obtain the least complex model that exhibits the best possible fitting.

## 2 Rudiments of the proposed approach

### 2.1 The EM algorithm

Let us consider an estimation problem and its corresponding log-likelihood function defined as *ℓ*(***θ***) = log *p*(***y***|***θ***), where *p*(***y***|***θ***) is the likelihood function, $\theta \in R^{p}$, and $y\in R^{N}$. Denoting the *complete data* by ***z*** ∈ Ω(***y***), and using Bayes’ theorem, we can obtain:

$$\begin{array}{c} \ell (\theta )=\text{log}\;p(y|\theta )=\text{log}\;p(z|\theta )-\text{log}\;p(z|y,\theta ). \end{array}$$ (1)

Let us assume that at the *i*th iteration we have the estimate ${\hat{\theta }}^{\left(i\right)}$. By integrating at both sides of (1) with respect to $p(z|y,{\hat{\theta }}^{\left(i\right)})$ we obtain $\ell \left(\theta \right)=Q\left(\theta ,{\hat{\theta }}^{\left(i\right)}\right)-H\left(\theta ,{\hat{\theta }}^{\left(i\right)}\right)$, where

$$Q(\theta ,{\hat{\theta }}^{\left(i\right)})=\int_{\Omega (y)}\text{log}\;p\left(z|\theta \right)p\left(z|y,{\hat{\theta }}^{\left(i\right)}\right)dz,$$ (2)

$$H(\theta ,{\hat{\theta }}^{\left(i\right)})=\int_{\Omega (y)}\text{log}\;p\left(z|y,\theta \right)p\left(z|y,{\hat{\theta }}^{\left(i\right)}\right)dz.$$ (3)

Using Jensen’s inequality [31], it is possible to show that for any value of ***θ***, the function $H(\theta ,{\hat{\theta }}^{\left(i\right)})$ is decreasing. Hence, the optimization is only carried out on the auxiliary function $Q(\theta ,{\hat{\theta }}^{\left(i\right)})$ because, by maximizing $Q(\theta ,{\hat{\theta }}^{\left(i\right)})$, the new parameter ${\hat{\theta }}^{\left(i+1\right)}$ is such that the likelihood function increases (see e.g. [5, 33]).

In general, the EM method can be summarised as follows:

E-step: Compute the expected value of the joint *likelihood function* for the *complete data* (measurements and hidden variables) based on a given parameter estimate, ${\hat{\theta }}^{\left(i\right)}$. Thus, we have (see e.g. [5]):
  $$\begin{array}{c} Q\left(\theta ,{\hat{\theta }}^{\left(i\right)}\right)=E\left[\;\text{log}\;p\left(z|\theta \right)|y,{\hat{\theta }}^{\left(i\right)}\;\right], \end{array}$$ (4)
M-step: Maximize the function $Q(\theta ,{\hat{\theta }}^{\left(i\right)})$ (4), with respect to ***θ***:
  $$\begin{array}{c} {\hat{\theta }}^{\left(i+1\right)}=\text{arg}\;\max_{\theta }Q\left(\theta ,{\hat{\theta }}^{\left(i\right)}\right). \end{array}$$ (5)

This succession of estimates converges to a stationary point of the *log-likelihood* function [34].

### 2.2 The MM algorithm

The idea behind the MM algorithm [7] is to construct a surrogate function $g(\theta ,{\hat{\theta }}^{\left(i\right)})$, that majorizes (for minimization problems) or minorizes (for maximization problems) a given cost functions *f*(***θ***) [7] at ${\hat{\theta }}^{\left(i\right)}$ such that,

$$\begin{array}{c} \begin{array}{cc} f\left(\theta \right)\leq g\left(\theta ,{\hat{\theta }}^{\left(i\right)}\right) & \;\text{for}\;\text{minimization}\;\text{problems,}\;\text{or}\\ f\left(\theta \right)\geq g\left(\theta ,{\hat{\theta }}^{\left(i\right)}\right) & \;\text{for}\;\text{maximization}\;\text{problems,}\;\text{and}\\ f\left(\theta \right)=g\left(\theta^{\left(i\right)},\theta^{\left(i\right)}\right), \end{array} \end{array}$$

where ${\hat{\theta }}^{\left(i\right)}$ is an estimate of ***θ***. Then, the surrogate function is iteratively optimized until convergence. Hence, for maximizing *f*(***θ***) we have [35]

$$\begin{array}{c} {\hat{\theta }}^{\left(i+1\right)}=\text{arg}\;\max_{\theta }g\left(\theta ,{\hat{\theta }}^{\left(i\right)}\right). \end{array}$$ (6)

For the construction of the surrogate function, popular techniques include the second order Taylor approximation, the quadratic upper bound principle and Jensen’s inequality for convex functions, see, e.g., [35].

**Remark 1**. *The iterative strategy utilized in the MM algorithm converges to a local optimum since*

$$\begin{array}{c} f\left({\hat{\theta }}^{\left(i+1\right)}\right)\geq g\left({\hat{\theta }}^{\left(i+1\right)},{\hat{\theta }}^{\left(i\right)}\right)\geq g\left({\hat{\theta }}^{\left(i\right)},{\hat{\theta }}^{\left(i\right)}\right)=f\left({\hat{\theta }}^{\left(i\right)}\right). \end{array}$$

### 2.3 Data augmentation in inference problems

Data augmentation algorithms are based on the construction of the *augmented data* and its many-to-one mapping Ω(*y*). This *augmented data* is assumed to describe a model from which the observed data *y* is obtained via marginalization [36]. That is, a system with a likelihood function *p*(*y*|***θ***) can be understood to arise from

$$\begin{array}{c} p(y|\theta )=\int p(y,x|\theta )dx, \end{array}$$ (7)

where the *augmented data* corresponds to (*y*, *x*) and *x* is the *latent data* [6, 36]. This idea has been utilized for supervised learning [37] and the development of the *Bayesian Lasso* [38], to mention a few examples. In those problems, the Laplace distribution is expressed as a two-level hierarchical-Bayes model. This equivalence is obtained from the representation of the Laplace distribution as a VMGM:

$$\begin{array}{c} \frac{a}{2}e^{-a|\theta |}=\int_{0}^{\infty }\underset{p(\theta |\lambda )}{\underset{︸}{\frac{1}{\sqrt{2\pi \lambda }}e^{-\theta^{2}/\left(2\lambda \right)}}}\underset{p(\lambda )}{\underset{︸}{\frac{a^{2}}{2}e^{-a^{2}\lambda /2}}}d\lambda . \end{array}$$ (8)

In fact, there are several pdf’s than can be expressed as VMGMs, as shown in Table 1 [22], where *g*(*θ*) is the penalty term that can be expressed as a pdf. In addition, in [26] it was developed an early version of the methodology presented in this paper, exploring the estimation of a sparse parameter vector utilizing the *ℓ*_*q*_-(pseudo)norm, with 0 < *q* < 1.

**Table 1:** Selection of mean-variance mixture representations for penalty functions. $p\left(\theta \right)\;=\;\int_{0}^{\infty }N_{\theta }\left(\mu \;+\;\lambda u,\tau^{2}s^{2}\lambda \right)p\left(\lambda \right)d\lambda$ .

Penalty function	*g*(*θ*)	*u*	*μ*	*p*(λ)
Ridge	(*θ*/*τ*)^2^	0	0	λ = 1
Lasso	|*θ*/*τ*|	0	0	Exponential
Bridge	|*θ*/*τ*|^*α*^	0	0	Stable
Generalized Double-Pareto	$\left[\frac{\left(1+\alpha \right)}{\tau }]\text{log}(1+\frac{\left|\theta \right|}{\left(\alpha \tau \right)}\right)$	0	0	Exp-Gamma

## 3 A systematic approach to construct surrogate functions for a class of inference problems

Here, we consider a general optimization cost defined as:

$$\begin{array}{c} V\left(\theta \right)=\int_{\Omega (y)}K\left(z,\theta \right)d\mu \left(z\right), \end{array}$$ (9)

where ***θ*** is a parameter vector, ***y*** is a given data (i.e. measurements), ***z*** is the *complete data* (comprised of the *observed data* ***y*** and the *hidden variables* (unobserved data), Ω(***y***) is a mapping from the sample space of ***z*** to the sample space of ***y***, *K*(⋅, ⋅) is a (positive) kernel function, and *μ*(⋅) is a measure, see e.g [31]. The definition in (9) is based on the definition of the auxiliary function Q in the EM algorithm [5], where it is assumed throughout the paper that there is a mapping that relates the *not observed data* to the *observed data*, and that the *complete data* lies in Ω(***y***) [5]. Notice that in (9) the kernel function may not be a pdf. However, several functions can be expressed in terms of a pdf. The most common cases are Gaussian kernels (yielding VMGMs) [23] and Laplace kernels (yielding Laplace mixtures) [39].

**Remark 2**. *Notice that, as explained in Section 1, once the hidden data has been selected, the data augmentation procedure comes with the definition of* V(θ) *in* (9). *From here, we follow the systematic nature of the EM and MM algorithms in terms of the iterative nature of the technique*.

### 3.1 Constructing the surrogate function

Since we are considering the optimization of the function V(θ), we can also consider the optimization of the function

$$\begin{array}{c} J(\theta )=\text{log}\;V(\theta ). \end{array}$$ (10)

Without modifying the cost function in (10), we can multiply and divide by the logarithm of the kernel function, obtaining:

$$\begin{array}{c} J\left(\theta \right)=\text{log}\;V\left(\theta \right)=\text{log}\;V\left(\theta \right)\frac{\text{log}\;K(z,\theta )}{\text{log}\;K(z,\theta )}=\text{log}\;K\left(z,\theta \right)-\text{log}\;\frac{K(z,\theta )}{V(\theta )}. \end{array}$$ (11)

Let us assume that at the *i*th iteration we have the estimate ${\hat{\theta }}^{\left(i\right)}$. Then, we can multiply by $\frac{K(z,{\hat{\theta }}^{\left(i\right)})}{V({\hat{\theta }}^{\left(i\right)}}$ and integrate on both sides of (11) with respect to *dμ*(***z***), obtaining:

$$\begin{array}{ll} J\left(\theta \right) & =\int_{\Omega \left(y\right)}\text{log}\;V\left(\theta \right)\frac{K\left(z,{\hat{\theta }}^{\left(i\right)}\right)}{V\left({\hat{\theta }}^{\left(i\right)}\right)}d\mu \left(z\right)=\text{log}\;V\left(\theta \right)\\ & =Q\left(\theta ,{\hat{\theta }}^{\left(i\right)}\right)-H\left(\theta ,{\hat{\theta }}^{\left(i\right)}\right). \end{array}$$ (12)

where:

$$Q(\theta ,{\hat{\theta }}^{\left(i\right)})=\int_{\Omega (y)}\text{log}\;\left[K\left(z,\theta \right)\right]\frac{K(z,{\hat{\theta }}^{\left(i\right)})}{V({\hat{\theta }}^{\left(i\right)})}d\mu \left(z\right),$$ (13)

$$H(\theta ,{\hat{\theta }}^{\left(i\right)})=\int_{\Omega (y)}\text{log}\;[\frac{K(z,\theta )}{V(\theta )}]\frac{K(z,{\hat{\theta }}^{\left(i\right)})}{V({\hat{\theta }}^{\left(i\right)})}d\mu \left(z\right),$$ (14)

are auxiliary functions. As in the EM algorithm, for any ***θ***, and using Jensen’s inequality [31], we have:

$$\begin{array}{ll} H\left(\theta ,{\hat{\theta }}^{\left(i\right)}\right)\;-H\left({\hat{\theta }}^{\left(i\right)},{\hat{\theta }}^{\left(i\right)}\right)\; & =\int_{\Omega (y)}\;\;\;\text{log}\;\;\;[\frac{K(z,\theta )}{V(\theta )}]\;\;\frac{K(z,{\hat{\theta }}^{\left(i\right)})}{V({\hat{\theta }}^{\left(i\right)})}d\mu \left(z\right)\\ & -\int_{\Omega (y)}\text{log}\;[\frac{K(z,{\hat{\theta }}^{\left(i\right)})}{V({\hat{\theta }}^{\left(i\right)})}]\frac{K(z,{\hat{\theta }}^{\left(i\right)})}{V({\hat{\theta }}^{\left(i\right)})}d\mu \left(z\right)\\ & =\int_{\Omega (y)}\text{log}\;[\frac{K\left(z,\theta \right)V\left({\hat{\theta }}^{\left(i\right)}\right)}{V\left(\theta \right)K\left(z,{\hat{\theta }}^{\left(i\right)}\right)}]\frac{K(z,{\hat{\theta }}^{\left(i\right)})}{V({\hat{\theta }}^{\left(i\right)})}d\mu \left(z\right)\\ & \leq \text{log}\;\int_{\Omega (y)}\frac{K(z,\theta )}{V(\theta )}d\mu \left(z\right)\\ & =0. \end{array}$$ (15)

Hence, for any value of ***θ***, the function $H(\theta ,{\hat{\theta }}^{\left(i\right)})$ in (14) is a decreasing function.

**Remark 3**. *The kernel function K*(***z***, ***θ***) *satisfies the standing assumption K*(***z***, ***θ***) > 0 *since the proposed scheme is built, among others, on the logarithm of the kernel function K*(***z***, ***θ***). *The definition of the kernel function in* (9) *allows for kernels that are not pdf’s. On the other hand, some kernels may correspond to a scaled version of a pdf. In that sense, for the cost function in* (9) *we can define a new kernel and a new measure as*

$$\begin{array}{c} \bar{K}\left(z,\theta \right)=\frac{K(z,\theta )}{\int K(z,\theta )d\theta },\;\;d\bar{\mu }\left(z\right)=(\int K(z,\theta )d\theta )d\mu \left(z\right), \end{array}$$

$$\Rightarrow V\left(\theta \right)=\int_{\Omega (y)}\bar{K}\left(z,\theta \right)d\bar{\mu }\left(z\right).$$

**Remark 4**. *In the proposed methodology, it is possible to optimize the surrogate function defined by*

$$\begin{array}{c} \bar{Q}\left(\theta ,{\hat{\theta }}^{\left(i\right)}\right)=\int_{\Omega (y)}\text{log}\;\left[K\left(z,\theta \right)\right]K(z,{\hat{\theta }}^{\left(i\right)})d\mu \left(z\right), \end{array}$$ (16)

*since* $V({\hat{\theta }}^{\left(i\right)})$ *in* (13) *does not depend on the parameter* ***θ***. *Thus, the proposed method corresponds to a variation of the EM algorithm that is not limited to probability density functions (e.g. the likelihood function) for solving ML and MAP estimation problems. Instead, our version considers general measures (μ*(***z***)*)*, *where the mapping over the measurement data* Ω(***y***) *is a given set*.

The idea behind using a surrogate function is to obtain a simpler algorithm for the optimization of the objective function when compared to the original optimization problem. This can be achieved iteratively if the Fisher Identity for the surrogate function and the objective function is satisfied. That is,

$$\frac{\partial }{\partial \theta }J\left(\theta \right){\left|{}_{\theta ={\hat{\theta }}^{\left(i\right)}}=\frac{\partial }{\partial \theta }Q\left(\theta ,{\hat{\theta }}^{\left(i\right)}\right)\right|}_{\theta ={\hat{\theta }}^{\left(i\right)}}.$$ (17)

**Lemma 1**. *For the class of objective functions in* (9), *the surrogate function* $Q(\theta ,{\hat{\theta }}^{\left(i\right)})$ *in* (13) *satisfies the Fisher identity defined in* (17).

*Proof*. From (12) we have:

$$\frac{\partial }{\partial \theta }J\left(\theta \right){|}_{\theta ={\hat{\theta }}^{\left(i\right)}}=\frac{\partial }{\partial \theta }Q\left(\theta ,{\hat{\theta }}^{\left(i\right)}\right){|}_{\theta ={\hat{\theta }}^{\left(i\right)}}-\frac{\partial }{\partial \theta }H\left(\theta ,{\hat{\theta }}^{\left(i\right)}\right){|}_{\theta ={\hat{\theta }}^{\left(i\right)}}.$$

Next, let us consider the gradient of the auxiliary function $H(\theta ,{\hat{\theta }}^{\left(i\right)})$:

$$\begin{array}{ll} \frac{\partial }{\partial \theta }H\left(\theta ,{\hat{\theta }}^{\left(i\right)}\right)|\theta ={\hat{\theta }}^{\left(i\right)} & =\int_{\Omega \left(y\right)}{\left[\frac{K\left(z,\theta \right)}{V\left(\theta \right)}\right]}_{\theta ={\hat{\theta }}^{\left(i\right)}}^{-1}\frac{\partial }{\partial \theta }{\left[\frac{K\left(z,\theta \right)}{V\left(\theta \right)}\right]}_{\theta ={\hat{\theta }}^{\left(i\right)}}\frac{K\left(z,{\hat{\theta }}^{\left(i\right)}\right)}{V\left({\hat{\theta }}^{\left(i\right)}\right)}d\mu \left(z\right)\\ & =\int_{\Omega \left(y\right)}\frac{\partial }{\partial \theta }{\left[\frac{K\left(z,\theta \right)}{V\left(\theta \right)}\right]}_{\theta ={\hat{\theta }}^{\left(i\right)}}d\mu \left(z\right)=\frac{\partial }{\partial \theta }{\left[\underset{\Omega \left(y\right)}{\int }\frac{K\left(z,\theta \right)}{V\left(\theta \right)}d\mu \left(z\right)\right]}_{\theta ={\hat{\theta }}^{\left(i\right)}}\\ & =0. \end{array}$$

Hence, (17) holds.

**Remark 5**. *Note that the Fisher identity in Lemma 1 is well known in the EM-framework. However, we have specialized this result for the problem in this paper (i.e. when K*(***z***, ***θ***) *is not necessarily a probability density function)*

**Lemma 2**. *The surrogate function* $Q(\theta ,{\hat{\theta }}^{\left(i\right)})$ *in* (13) *can be utilized to obtain an adequate surrogate function that satisfies the properties in Mark’s approach in* (38)–(40).

*Proof*. Notice that in Mark’s approach the optimization problem corresponds to the minimization of the objective function. Hence, to maximize, we have $J\left(\theta \right)=-Q\left(\theta ,{\hat{\theta }}^{\left(i\right)}\right)+H\left(\theta ,{\hat{\theta }}^{\left(i\right)}\right)$. From (12) we can construct the surrogate functions $\tilde{Q}\left(\theta ,{\hat{\theta }}^{\left(i\right)}\right)$ and $\tilde{H}\left(\theta ,{\hat{\theta }}^{\left(i\right)}\right)$ since

$$\begin{array}{c} J\left(\theta \right)=-\underset{\tilde{Q}\left(\theta ,{\hat{\theta }}^{\left(i\right)}\right)}{\underset{︸}{(Q\left(\theta ,{\hat{\theta }}^{\left(i\right)}\right)-Q\left({\hat{\theta }}^{\left(i\right)},{\hat{\theta }}^{\left(i\right)}\right)+J\left({\hat{\theta }}^{\left(i\right)}\right)}}+\underset{\tilde{H}\left(\theta ,{\hat{\theta }}^{\left(i\right)}\right)}{\underset{︸}{(H\left(\theta ,{\hat{\theta }}^{\left(i\right)}\right)-Q\left({\hat{\theta }}^{\left(i\right)},{\hat{\theta }}^{\left(i\right)}\right)+J\left({\hat{\theta }}^{\left(i\right)}\right)}}. \end{array}$$ (18)

The function $\tilde{H}\left(\theta ,{\hat{\theta }}^{\left(i\right)}\right)$ satisfies $\tilde{H}\left(\theta ,{\hat{\theta }}^{\left(i\right)}\right)-\tilde{H}\left({\hat{\theta }}^{\left(i\right)},{\hat{\theta }}^{\left(i\right)}\right)$ ≥ 0, which implies that $J\left(\theta \right)\leq \tilde{Q}\left(\theta ,{\hat{\theta }}^{\left(i\right)}\right)$, satisfying (38). From $\tilde{Q}\left(\theta ,{\hat{\theta }}^{\left(i\right)}\right)=Q\left(\theta ,{\hat{\theta }}^{\left(i\right)}\right)-Q\left({\hat{\theta }}^{\left(i\right)},{\hat{\theta }}^{\left(i\right)}\right)+J\left({\hat{\theta }}^{\left(i\right)}\right)$ we can obtain $\tilde{Q}\left({\hat{\theta }}^{\left(i\right)},{\hat{\theta }}^{\left(i\right)}\right)=J\left({\hat{\theta }}^{\left(i\right)}\right)$, satisfying (39). Finally, given that the auxiliary function $\tilde{H}\left(\theta ,{\hat{\theta }}^{\left(i\right)}\right)$ satisfies (17), $\tilde{Q}\left(\theta ,{\hat{\theta }}^{\left(i\right)}\right)$ satisfies (40).

**Remark 6**. *Since* $\frac{d}{d\theta }Q\left(\theta ,{\hat{\theta }}^{\left(i\right)}\right)=\frac{d}{d\theta }\tilde{Q}\left(\theta ,{\hat{\theta }}^{\left(i\right)}\right)$, *it is simpler to consider the function* $Q(\theta ,{\hat{\theta }}^{\left(i\right)})$ *instead of* $\tilde{Q}\left(\theta ,{\hat{\theta }}^{\left(i\right)}\right)$ *in penalized (regularized) and MAP estimation problems, as shown in* [26] *and* [27].

We summarize our proposed algorithm in Table 2.

**Table 2:** Proposed algorithm.

Step 1: Find a kernel that satisfies (9).
Step 2: *i* = 0.
Step 3: Obtain an initial guess ${\hat{\theta }}^{\left(i\right)}$.
Step 4: Compute $Q(\theta ,{\hat{\theta }}^{\left(i\right)})$ as in (13).
Step 5: Compute $\tilde{Q}\left(\theta ,{\hat{\theta }}^{\left(i\right)}\right)$.
Step 6: Incorporate $\tilde{Q}\left(\theta ,{\hat{\theta }}^{\left(i\right)}\right)$ in the optimization problem and solve.
Step 7: *i* = *i* + 1 and back to Step 4 until convergence.

### 3.2 Surrogate functions for inverse problems

The objective function in (9) can be understood as an integral equation [40], where the unknown function of the integral equation corresponds to the kernel *K*(***z***, ***θ***). In this kind of problems, samples from V(θ) are available, whilst *dμ*(***z***) is assumed known. Several problems can be posed as this kind of problems. In the following, we explain how to use the approach presented in this paper to solve different inverse problems that arise in the integral equation form.

#### 3.2.1 Stellar rotational velocity estimation

One of the many problems in Astronomy deals with is the estimation of rotational velocities of stars. This particular problem is of great importance, since it allows astronomers to describe and model the stars formation, their internal structure and evolution, as well as how they interact with other stars, see e.g. [19, 41, 42].

Modern telescopes allow for the measurement of the rotational velocities from the telescope point of view, that is, a projection of the true rotational velocity. This is modelled (spatially) as the convolution of the true rotational velocity pdf and a uniform distribution over the sphere (for more details see e.g. [19]):

$$\begin{array}{c} p\left(y|\sigma \right)=\int_{y}^{\infty }\frac{y}{x\sqrt{x^{2}-y^{2}}}p\left(x|\sigma \right)dx, \end{array}$$ (19)

where *p*(*y*|*σ*) is the uniform projected rotational velocity pdf and *p*(*x*|*σ*) is the true rotational velocity pdf to be estimated, and *σ* a hyperparameter. Thus, we can define the kernel function as *K*(*x*, *σ*) = *p*(*x*|*σ*) and $d\mu \left(x\right)=\frac{y}{x\sqrt{x^{2}-y^{2}}}dx$. This definition allows for the direct utilization of the expressions in (13) in order to estimate the parameter *σ* that defines the unknown rotational velocity pdf. We illustrate with one example in Section 6.1.

#### 3.2.2 Channel estimation in wireless communications

It is well known in the Communications community that the wireless channel corresponds to the superposition of different copies of the transmitted signal that have been reflected, refracted and scattered. Thus, those copies arrive at the receiver with different phase. Those components are referred to as *multipath components* [43]. On the other hand, it has been shown empirically that a good model for the multipath channel corresponds to either Rayleigh or Rice distributions [44]. However, there are cases when those distributions do not provide a good model for the channel. One of those cases corresponds to the presense of different channel models in the vicinity of the transmitter and the vicinity of the receiver. This situation ocurrs particularly in the so called *urban scenarios*, where the channel exhibits different behaviouirs in different places. For this scenario, an adequate model that takes into consideration different models and a transition from a *local distribution* and a *global* distribution is a continuous mixture of the form [18]

$$\begin{array}{c} p\left(x\right)=\int_{\Omega (x)}K\left(x,\sigma \right)d\mu \left(\sigma \right), \end{array}$$ (20)

where *K*(***x***, ***σ***) is a pdf in a local area, *μ*(***σ***) is a distribution of ***σ*** which depends on the path from transmitter to the local cluster, and ***σ*** is a vector in the parameter space. Our approach can be directly used in order to estimate the true nature of the channel when expressed as a mixture. This can be done since the four most common chanel distributions are Rayleigh, Rice, log-normal and Nakagami-*m* [45]. Hence, assuming that the local and global distribution families are known, the attainment of the auxiliary function $Q(\sigma ,{\hat{\sigma }}^{\left(i\right)})$ is straightforward and thus the ML estimate of ***σ***.

#### 3.2.3 Neutrino mass search in particle physics

In the Particle Physics community there is a plethora of works dealing with the estimation of masses of neutrinos, see e.g. [20] and the references therein. Among the methods that are generally used to determine the absolute masses of neutrinos we find the *β*-decay and the direct determination of neutrino mass, see e.g [21]. In *β*-decay methods, the neutrinos are analized based on their energy spectrums, where the measurements, corresponding to the observed *β*-spectra, are associated with an integral equation of the form [20, 21]

$$\begin{array}{c} F\left(U\right)=\int R\left(E\right)T\prime \left(E,U\right)dE+b, \end{array}$$ (21)

where *b* is a constant that represents the measurement noise, *R*(*E*) is the emitted *β*-spectra, and *T*′(*E*, *U*) is the impulse response of the equipment. Again, by regarding *T*′(*E*, *U*) as the kernel function and *R*(*E*)*dE* as a measure, the attainment of the auxiliary function Q is straightforward.

#### 3.2.4 Estimation of mixture distributions

Mixture distributions have been widely studied in the literature, particularly finite mixtures, see e.g. [46, 47]. Their representation can be expressed in a general fashion by the notation [48]

$$\begin{array}{c} p\left(y|\theta \right)=\int_{\Omega (y)}K\left(z,\theta \right)d\mu \left(z\right), \end{array}$$ (22)

where *K*(***z***, ***θ***) is a suitable function that may be either a pdf (for continuous random variables) or a probability function (for discrete random variables). The expression in (22) represents both the sum

$$\begin{array}{c} p\left(y|\theta \right)=\sum_{z_{j}\in \Omega \left(y\right)}K\left(z_{j},\theta \right), \end{array}$$ (23)

for a finite mixture, or the integral

$$\begin{array}{c} p\left(y|\theta \right)=\int_{\Omega (y)}K\left(z,\theta \right)dz, \end{array}$$ (24)

for an infinite mixture.

Our approach can be tailored for the estimation of the parameters of a finite mixture of the form

$$\begin{array}{c} p\left(y|\beta \right)=\prod_{k=1}^{N}\sum_{j=1}^{M}\lambda_{j}\phi_{j}\left(y_{k},\theta_{j}\right), \end{array}$$ (25)

where we have assumed that $p\left(y|\beta \right)=\prod_{k=1}^{N}p\left(y_{k}|\beta \right)$, $p\left(y|\beta \right)=\sum_{j=1}^{M}\lambda_{j}\phi_{j}\left(y_{k},\theta_{j}\right)$, and **λ** = [λ_1_,…, λ_*M*_] are the mixing weights, *ϕ*_*j*_(***y***, *θ*_*j*_) are the components densities parametrised by *θ*_*j*_. The kernel function defined in our approach can be utilized to represent *j*th component in the discrete mixture in (25) as

$$\begin{array}{c} K_{j}\left(z_{j},\beta_{j}\right)=\lambda_{j}\phi_{j}\left(y,\theta_{j}\right), \end{array}$$ (26)

where the ***β*** = [***β***_1_,…, ***β***_*M*_] is the vector of parameters to be estimated, with ***β***_*j*_ = [λ_*j*_, ***θ***_*j*_]. Notice that the dependence with respect to the variable ***z*** is implicit. Utilizing the expression derived in (13) we obtain the following E-step

$$\begin{array}{c} Q\left(\beta ,{\hat{\beta }}^{\left(i\right)}\right)=\sum_{j=1}^{M}\text{log}\;K_{j}\left(z_{j},\beta_{j}\right)\frac{K_{j}\left(z_{j},{\hat{\beta }}_{j}^{\left(i\right)}\right)}{\sum_{j=1}^{M}K_{j}\left(z_{j},{\hat{\beta }}_{j}^{\left(i\right)}\right)}. \end{array}$$ (27)

Notice that, as shown in [49], the auxiliary function $Q(\beta ,{\hat{\beta }}^{\left(i\right)})$ in (27) is the same that we obtain when we consider that the data are fully categorized, i.e. each *y*_*k*_, *k* = 1,…, *N*, is assumed to be drawn from only one distribution of the mixture. This assumption yields a data augmentation problem that is solved using the EM algorithm [46].

**Remark 7**. *If we consider a combination of infinite mixtures and finite mixtures of the form*

$$\begin{array}{c} p\left(y|\beta \right)=\prod_{k=1}^{N}\int p\left(y_{k}|x_{k}\right)p\left(x_{k}|\beta \right)dx_{k}, \end{array}$$ (28)

*with*

$$\begin{array}{c} p\left(x_{k}|\beta \right)=\sum_{j=1}^{M}\lambda_{j}\phi_{j}\left(x_{k},\theta_{j}\right), \end{array}$$ (29)

*we can utlize the same approach described here to solve the problem of estimating the parameters in* (28), ***β***. *In this case, the jth kernel is defined as* [50]

$$\begin{array}{c} K_{j}\left(x_{k},\beta_{j}\right)=\lambda_{j}\phi \left(x_{k};\theta_{j}\right), \end{array}$$ (30)

*and measure*

$$\begin{array}{c} d\mu \left(x_{k}\right)=p\left(y_{k}|x_{k}\right)dx_{k}. \end{array}$$ (31)

*Then, the log-likelihood function can be expressed as*

$$\begin{array}{c} \ell_{N}\left(\beta \right)=\sum_{k=1}^{N}\text{log}\;[V_{k}\left(\beta \right)], \end{array}$$ (32)

*with*

$$\begin{array}{c} V_{k}\left(\beta \right)=\sum_{j=1}^{M}\int_{-\infty }^{\infty }K\left(x_{k},\beta_{j}\right)d\mu \left(x_{k}\right). \end{array}$$ (33)

*This choice of functions leads to the direct implementation of our proposal, from which it is obtained*

$$\begin{array}{c} Q_{k}\left(\beta ,{\hat{\beta }}^{\left(i\right)}\right)=\sum_{j=1}^{M}\int_{-\infty }^{\infty }\text{log}\;[K(x_{k},\beta_{j})]\frac{K(x_{k},{\hat{\beta }}_{j}^{\left(i\right)})}{V_{k}\left({\hat{\beta }}^{\left(i\right)}\right)}d\mu \left(x_{k}\right), \end{array}$$ (34)

*and the ML estimator can be locally obtained from*

$$\begin{array}{c} \bar{Q}\left(\beta ,{\hat{\beta }}^{\left(i\right)}\right)=\sum_{k=1}^{N}Q_{k}\left(\beta ,{\hat{\beta }}^{\left(i\right)}\right), \end{array}$$ (35)

$$\begin{array}{c} {\hat{\beta }}^{\left(i+1\right)}=\arg\max_{\beta }\;\bar{Q}\left(\beta ,{\hat{\beta }}^{\left(i\right)}\right). \end{array}$$ (36)

## 4 Marks’ approach for constrained optimization

### 4.1 Constrained problems in statistical inference

Statistical Inference and System Identification techniques include a variety of methods that can be used in order to obtain a model of a system from data. Classical methods, such as *Least Squares*, ML, MAP [51], *Prediction Error Method*, *Instrumental Variables* [52], and *Stochastic Embedding* [53] have been considered in the literature for such task. However, the increasing complexity of modern system models has motivated researchers to revisit and reconsider those techniques for some problems. This has resulted in the incorporation of constraints and penalties, yielding an often more complicated optimization problem. For instance, it has been shown that the incorporation of linear equality constraints may improve the accuracy of the parameter estimates, see e.g [54]. On the other hand, the incorporation of regularization terms (or penalties) also improves the accuracy of the estimates, reducing the effect of noise and eliminating spurious local minima [55]. Regularization can be mainly incorporated in two ways: by adding regularizing constraints (a penalty function) or by including a probability density function (pdf) as a prior distribution for the parameters, see e.g. [27]. Another way to improve the estimation is by incorporating inequality constraints, where certain functions of the parameters may be required, for physical reasons amongst others, to lie between certain bounds [56]. From this point of view, it is possible to consider the classical methods with constraints or penalties, as in [53, 55–57].

Perhaps one of the most utilized approaches for penalized estimation (with complicated non-linear expressions) is the MM algorithm—for details on the MM algorithm see Section 2.2. This technique allows for the utilization of a surrogate function that is simple to handle, in terms of derivatives and optimization techniques, and that is, in turn, iteratively solved. However, its inequality constraint counterpart, here referred to as Marks’ approach [10], is not as well known as the MM algorithm. Moreover, there is no straightforward manner to obtain such surrogate function. In this paper we focus on a systematic way to obtain the corresponding surrogate function using Marks’ approach for a class of constraints.

### 4.2 Mark’s approach

The approach in [10] deals with inequality constraints by using a similar approach to the EM and MM algorithms. The basic idea is, again, to generate a surrogate function that allows for an iterative procedure whose optimum value is the optimum value of the original optimization problem.

Let us consider the following constrained optimization problem:

$$\begin{array}{cc} \theta *= & \;\text{arg}\;\underset{\theta }{\text{min}}\;f\left(\theta \right)\\ \text{s.}\;\text{t.}\; & \;\;g(\theta )\leq 0, \end{array}$$ (37)

where *f*(***θ***) is the objective function and *g*(***θ***) encodes the constraint of the optimization problem. In particular, let us focus on the case where *g*(***θ***) is not a convex function. This implies that the optimization problem cannot be solved directly using standard techniques, such as quadratic programming or fractional programming. This difficulty can be overcome by utilizing a surrogate function $Q(\theta ,{\hat{\theta }}^{\left(i\right)})$ at a given estimate ${\hat{\theta }}^{\left(i\right)}$, such that

$$g(\theta )\leq Q(\theta ,{\hat{\theta }}^{\left(i\right)})$$ (38)

$$g({\hat{\theta }}^{\left(i\right)})=Q({\hat{\theta }}^{\left(i\right)},{\hat{\theta }}^{\left(i\right)})$$ (39)

$$\frac{d}{d\theta }g\left(\theta \right){|}_{\theta ={\hat{\theta }}^{\left(i\right)}}=\frac{d}{d\theta }Q\left(\theta ,{\hat{\theta }}^{\left(i\right)}\right){|}_{\theta ={\hat{\theta }}^{\left(i\right)}}$$ (40)

Provided the above properties are satisfied, then the following approximation of (37):

$$\begin{array}{l} \theta^{\left(i+1\right)}=\text{arg}\;\underset{\theta }{\text{min}}f\left(\theta \right)\\ \text{s}.\;\text{t}.\;Q\left(\theta ,{\hat{\theta }}^{\left(i\right)}\right)\leq 0, \end{array}$$ (41)

iteratively converges to the solution of the optimization problem (37). As shown in [10], the optimization problem in (41) is equivalent to the original problem (37), since the solution of (41) converges to a point that satisfies the Karush-Kuhn-Tucker conditions of the original optimization problem.

**Remark 8**. *Mark’s approach can be considered as a generalization of the MM algorithm, since the latter can be derived (for a broad class of problems) from the former. Let us consider the following problem*:

$$\begin{array}{c} \theta *=\text{arg}\;\underset{\theta }{\text{min}}\;f\left(\theta \right). \end{array}$$ (42)

*Using the epigraph representation of* (42) [58], *we obtain the equivalent problem*

$$\begin{array}{l} \theta *=\text{arg}\;\underset{\theta }{\text{min}}\;t\\ s.\;t.\;f\left(\theta \right)\leq t, \end{array}$$ (43)

*Using Mark’s approach* (41), *we can iteratively find a local optimum of* (42) *via*

$$\begin{array}{l} \theta^{\left(i+1\right)}=\;\text{arg}\;\underset{\theta }{\text{min}}\;t\\ s.\;t.\;Q\left(\theta ,{\hat{\theta }}^{\left(i\right)}\right)\leq t, \end{array}$$ (44)

*where* $Q(\theta ,{\hat{\theta }}^{\left(i\right)})$ *in* (44) *is a surrogate function for f*(***θ***) *in* (42). *From the epigraph representation we then obtain*

$$\theta^{\left(i+1\right)}=\;\;\text{arg}\;\underset{\theta }{\text{min}}\;Q\left(\theta ,{\hat{\theta }}^{\left(i\right)}\right),$$ (45)

*which is the definition of the MM algorithm (see 2.2) for more details*.

## 5 A quadratic surrogate function for a class of kernels

In this section we focus on a special class of the kernel functions *K*(***z***, ***θ***). For this particular class, the following is satisfied:

$$\begin{array}{c} \frac{\partial }{\partial \theta }\text{log}\;\left[K\left(z,\theta \right)\right]=A\left(z\right)\theta +b, \end{array}$$ (46)

where **A**(***z***) is a matrix and **b** is a vector, both of adequate dimensions. Then, we have that

$$\begin{array}{l} \frac{\partial }{\partial \theta }Q\left(\theta ,{\hat{\theta }}^{\left(i\right)}\right)=\int_{\Omega \left(y\right)}\left[\text{A}\left(z\right)\theta +b\right]\frac{K\left(z,{\hat{\theta }}^{\left(i\right)}\right)}{V\left({\hat{\theta }}^{\left(i\right)}\right)}d\mu \left(z\right)\\ =\left[\int_{\Omega \left(y\right)}\text{A}\left(z\right)\frac{K\left(z,{\hat{\theta }}^{\left(i\right)}\right)}{V\left({\hat{\theta }}^{\left(i\right)}\right)}d\mu \left(z\right)\right]\theta +b\int_{\Omega \left(y\right)}\frac{K\left(z,{\hat{\theta }}^{\left(i\right)}\right)}{V\left({\hat{\theta }}^{\left(i\right)}\right)}d\mu \left(z\right)\\ =R\theta +b. \end{array}$$ (47)

**Remark 9**. *Notice that the previous expression is linear with respect to* ***θ***. *This implies that the function* $Q(\theta ,{\hat{\theta }}^{\left(i\right)})$ *is quadratic with respect to the parameter vector* ***θ***.

From the Fisher Identity in (17) we have that

$$\begin{array}{c} \frac{\partial }{\partial \theta }J\left(\theta \right){|}_{\theta ={\hat{\theta }}^{\left(i\right)}}=R{\hat{\theta }}^{\left(i\right)}+b, \end{array}$$ (48)

from which we can solve for **R** in some cases. In other cases, the matrix **R** can also be computed using Monte Carlo algorithms. In particular, if **A**(***z***) is a diagonal matrix, then **R** is also a diagonal matrix defined by **R** = diag[*r*_1_, *r*_2_,…]. Thus, we have

$$\begin{array}{c} \frac{\partial }{\partial \theta_{k}}J\left(\theta \right){|}_{\theta ={\hat{\theta }}^{\left(i\right)}}\;\;\;=r_{k}{\hat{\theta }}_{i}^{\left(i\right)}\;+b_{k}\Rightarrow r_{k}\;=\;\frac{\frac{\partial }{\partial \theta_{k}}J\left(\theta \right){|}_{\theta ={\hat{\theta }}^{\left(i\right)}}-b_{k}}{{\hat{\theta }}_{k}^{\left(i\right)}}, \end{array}$$

where *θ*_*i*_ is the *i*th component of the parameter vector ***θ***, ${\hat{\theta }}_{i}^{\left(i\right)}$ is the *i*th component of the estimate ${\hat{\theta }}^{\left(i\right)}$, *r*_*i*_ is the *i*th element of the diagonal of **R**, and *b*_*i*_ is the *i*th element of the vector **b**. Hence, when optimizing the auxiliary function $Q(\theta ,{\hat{\theta }}^{\left(i\right)})$ we obtain

$$\begin{array}{c} \frac{\partial }{\partial \theta }Q\left(\theta ,{\hat{\theta }}^{\left(i\right)}\right)=[\begin{array}{ccc} r_{1} & & \\ & r_{2} & \\ & & \ddots \end{array}]\theta +b=0\;\Rightarrow \;{\hat{\theta }}_{k}^{\left(i+1\right)}=-\frac{b_{k}}{r_{k}}. \end{array}$$

Equivalently,

$$\begin{array}{c} {\hat{\theta }}^{\left(i+1\right)}=R^{-1}b. \end{array}$$ (49)

This implies that in our approach, it is not necessary to obtain the auxiliary function Q and optimize it. Instead, by computing **R** and **b** at every iteration, the new estimate can be obtained.

**Remark 10**. *The computation of the matrix* ***R*** *can be cumbersome when the matrix* ***A***(***z***) *is not diagonal. In those cases, the integral that defines* ***R*** *can be computed utilizing Markov Chain Monte Carlo, quasi-Monte Carlo* [59] *or quadrature methods* [60].

The class defined in (46) arises naturally when dealing with VMGM, because the corresponding kernel function is normal and, thus, its logarithm is a quadratic function.

In particular, the utilization of VMGM encompasses different expressions commonly used for parameter estimation. Indeed, we have:

(i) Lasso: The Lasso [61], expressed as a Laplace pdf, is represented by a VMGM [38], with *p*(***θ***|***z***) a zero-mean Gaussian distribution (of iid terms) as the kernel and *p*(***z***) an exponential distribution with parameter *γ*^2^/2. That is,
  $$\begin{array}{c} p\left(\theta \right)=\prod_{j=1}^{p}\frac{\gamma }{2}e^{-\gamma |\theta_{j}|}=\prod_{j=1}^{p}\int N_{\theta_{j}}\left(0,z_{j}\right)(\frac{\gamma^{2}}{2}e^{-\frac{\gamma^{2}}{2}z_{j}})dz_{j}. \end{array}$$ (50)
(ii) Elastic-Net: The Elastic-Net penalty [62] is interpreted as a pdf if it corresponds to the product of two pdf’s, a Laplacian (as in the Lasso case) and a Gaussian pdf. In this sense, by grouping those pdf’s, we obtain [63]
  $$\begin{array}{c} p\left(\theta \right)=k_{EN}\prod_{j=1}^{p}\int_{1}^{\infty }N_{\theta_{j}}(0,\frac{\left(\lambda_{j}-1\right)}{\lambda_{j}\kappa })p\left(\lambda_{j}\right)d\lambda_{j}, \end{array}$$ (51)
  with $p\left(\lambda_{j}\right)\propto \sqrt{\frac{1}{\lambda_{j}-1}}e^{-\frac{1}{8}\frac{{\left(1-\kappa \right)}^{2}}{\kappa }\lambda_{j}}$.
(iii) Group-Lasso: The Group-Lasso penalty is obtained via VMGM-representation as
  $$\begin{array}{c} p\left(\theta \right)=k_{GL}\prod_{j=1}^{\text{p}}\int_{0}^{\infty }N_{\theta_{\text{g}}}\left(0,\frac{\lambda_{\text{g}}}{\gamma }{\text{I}}_{{\text{G}}_{\text{g}}}\right)\chi_{{\text{G}}_{\text{g}}+1}^{2}(\lambda_{\text{g}})d\lambda_{\text{g}}, \end{array}$$ (52)
  where $\chi_{l}^{2}$ is the chi-squared distribution with *l* degrees of freedom.

For the class of kernels here described, the proposed method for constructing surrogate functions can also be understood as part of sequential quadratic programming (SQP) methods [64] when, for instance, the above penalties are utilized as constraints in a constrained ML estimation problem. Indeed, the general case of equality and inequality-constrained minimization problems is defined as [65]:

$$\begin{array}{l} \theta *=\text{arg}\;\underset{\theta }{\text{min}}\;f\left(\theta \right)\\ \text{s}.\text{t}.\;\;h\left(\theta \right)=0,g\left(\theta \right)\leq 0, \end{array}$$ (53)

which is solved by iteratively defining quadratic functions that approximate the objective function and the inequality constraint around a current iterate ${\hat{\theta }}^{\left(i\right)}$. In the same way, our proposal generates an algorithm with quadratic surrogate functions, where an auxiliary function $\tilde{Q}\left(\theta ,{\hat{\theta }}^{\left(i\right)}\right)$ in (18) must be constructed for *f*(***θ***) and/or *g*(***θ***) in (53).

## 6 Numerical examples

In this section, we illustrate our proposed algorithm with two numerical examples.

### 6.1 Example 1: Estimation of the distribution of stellar rotational velocities

A common model for *p*(*x*|*σ*) found in the Astronomy literature is the Maxwellian distribution (see e.g. [19, 66])

$$\begin{array}{c} p\left(x|\sigma \right)=\sqrt{\frac{2}{\pi }}\frac{1}{\sigma^{3}}x^{2}e^{-x^{2}/\left(2\sigma^{2}\right)}. \end{array}$$ (54)

In practice, the measurements correspond to realizations of *p*(*y*|*σ*) [19], from which the likelihood function can be defined as:

$$\begin{array}{c} p\left(y|\sigma \right)=\prod_{k=1}^{N}p\left(y_{k}|\sigma \right), \end{array}$$ (55)

where **y** = [*y*_1_,…, *y*_*N*_]^*T*^,

$$\begin{array}{c} p\left(y_{k}|\sigma \right)=\int_{y_{k}}^{\infty }\frac{y_{k}}{x_{k}\sqrt{x_{k}^{2}-y_{k}^{2}}}p\left(x_{k}|\sigma \right)dx_{k}, \end{array}$$

*x*_*k*_ is Maxwellian distributed, and *N* is the number of measurement points. Hence, the log-likelihood function can be expressed as:

$$\begin{array}{c} \ell \left(\sigma \right)=\sum_{k=1}^{N}\text{log}\;[\int_{y_{k}}^{\infty }\;\;\frac{y_{k}}{x_{k}\sqrt{x_{k}^{2}-y_{k}^{2}}}p\left(x_{k}|\sigma \right)dx_{k}]. \end{array}$$ (56)

If we define the complete data ***z*** = (**x**, **y**), the kernel function *K*(⋅, ⋅) and the measure *μ*(⋅) in (9) can be defined as

$$\begin{array}{c} K\left(x_{k},\sigma \right)=p\left(x_{k}|\sigma \right)=\sqrt{\frac{2}{\pi }}\frac{x_{k}^{2}}{\sigma^{3}}e^{-x_{k}^{2}/\left(2\sigma^{2}\right)}, \end{array}$$ (57)

and

$$\begin{array}{c} d\mu \left(x_{k},y_{k}\right)=\frac{y_{k}}{x_{k}\sqrt{x_{k}^{2}-y_{k}^{2}}}dx_{k}. \end{array}$$ (58)

Then, the log-likelihood function in (56) can be written as:

$$\begin{array}{c} \ell \left(\sigma \right)=\sum_{k=1}^{N}\text{log}\;[V_{k}\left(\sigma \right)], \end{array}$$ (59)

with

$$\begin{array}{c} V_{k}\left(\sigma \right)=\int_{y_{k}}^{\infty }K\left(x_{k},\sigma \right)d\mu \left(x_{k},y_{k}\right), \end{array}$$ (60)

Thus, the ML estimator is obtained from:

$$\begin{array}{c} {\hat{\sigma }}_{\text{ML}}=\arg\max_{\sigma }\;\sum_{k=1}^{N}\text{log}\;V_{k}\left(\sigma \right). \end{array}$$ (61)

Since the parameter that is needed to be estimated is part of the convolution in (19), the optimization problem in (61) cannot be solved in a straightforward manner. Instead, we utilize the re-interpretation of the EM algorithm that we propose for solving (61).

First, notice that from the surrogate function $Q(\sigma ,{\hat{\sigma }}^{\left(i\right)})$ can be expressed as:

$$\begin{array}{c} Q\left(\sigma ,{\hat{\sigma }}^{\left(i\right)}\right)=\sum_{k=1}^{N}Q_{k}\left(\sigma ,{\hat{\sigma }}^{\left(i\right)}\right), \end{array}$$ (62)

with

$$\begin{array}{c} Q_{k}\left(\sigma ,{\hat{\sigma }}^{\left(i\right)}\right)=\int_{y_{k}}^{\infty }\text{log}\;\left(K\left(x_{k},\sigma \right)\right)\frac{K(x_{k},{\hat{\sigma }}^{\left(i\right)})}{V_{k}\left({\hat{\sigma }}^{\left(i\right)}\right)}d\mu \left(x_{k},y_{k}\right). \end{array}$$ (63)

For convenience, we can differentiate the auxiliary function $Q(\sigma ,{\hat{\sigma }}^{\left(i\right)})$ in (62) with respect to 1/*σ* obtaining:

$$\begin{array}{c} \frac{\partial Q(\sigma ,{\hat{\sigma }}^{\left(i\right)})}{\partial (1/\sigma )}=\sum_{k=1}^{N}\int_{y_{k}}^{\infty }[3\sigma -\frac{x_{k}^{2}}{\sigma }]\;\;\frac{K(x_{k},{\hat{\sigma }}^{\left(i\right)})}{V_{k}\left({\hat{\sigma }}^{\left(i\right)}\right)}d\mu \left(x_{k},y_{k}\right). \end{array}$$ (64)

Then, equating to zero and solving for *σ* we finally obtain

$$\begin{array}{c} {\hat{\sigma }}^{\left(i+1\right)}=\sqrt{\frac{S(\text{y},{\hat{\sigma }}^{\left(i\right)})}{3N}}, \end{array}$$ (65)

where

$$\begin{array}{c} S\left(\text{y},{\hat{\sigma }}^{\left(i\right)}\right)=\sum_{t=1}^{N}\int_{y_{k}}^{\infty }x_{k}^{2}\frac{K(x_{k},{\hat{\sigma }}^{\left(i\right)})}{V_{k}\left({\hat{\sigma }}^{\left(i\right)}\right)}d\mu \left(x_{k},y_{k}\right). \end{array}$$ (66)

In Table 3 we summarized the specialisation of our proposed algorithm for this example.

**Table 3:** Proposed algorithm for Maxwellian distribution estimation in Example 1.

Step 1: *i* = 0.
Step 2: Obtain an initial guess ${\hat{\sigma }}^{\left(i\right)}$.
Step 3: Compute the integral given by (66).
Step 4: Compute ${\hat{\sigma }}^{\left(i+1\right)}$ (65)
Step 5: *i* = *i* + 1 and back to Step 3 until convergence.

For the numerical simulation, we have considered the problem solved in [19], with the true dispersion parameter *σ*_0_ = 8. The measurement data **y** = [*y*_1_,…, *y*_*N*_] was generated using the *Slice Sampler* (see e.g. [67]) applied to (19). The simulation setup is as follows:

- The data length is given by *N* = 10000.
- The number of Monte Carlo (MC) simulations is 50.
- The stopping criterion is given by:
  $$\begin{array}{c} ∥{\hat{\sigma }}^{\left(i\right)}-{\hat{\sigma }}^{\left(i-1\right)}∥/∥{\hat{\sigma }}^{\left(i\right)}∥<10^{-6}, \end{array}$$
  or the maximum number of iterations of 100 has been reached.

**Fig 1:**
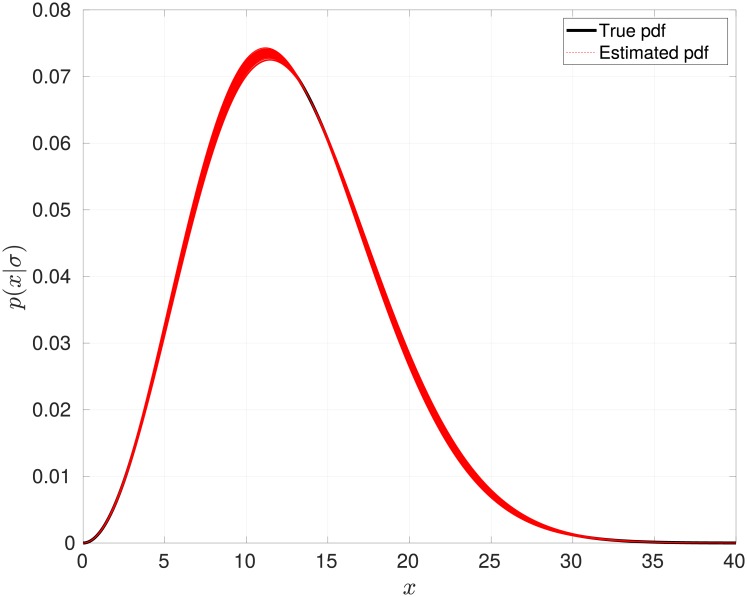
Estimated distribution for the stellar rotational velocity.

**Fig 2:**
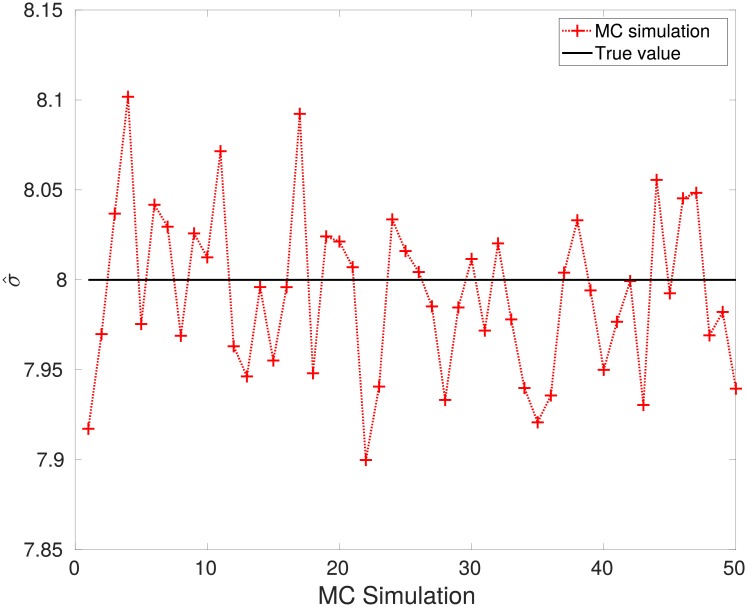
Convergence of the proposed approach to the global optimum.

The results are shown in Fig 1, were the estimated *p*(*x*|*σ*) for each MC simulation is shown. It is clear that the estimated Maxwellian distributions are very similar to the *true* density distribution. The mean value of the estimated parameter was $\hat{\sigma }=7.9920$. The estimation from each MC simulation is shown in Fig 2. It can be clearly seen that the estimated parameter $\hat{\sigma }$ is close to the *true* value.

### 6.2 Example 2: Channel estimation in wireless communications

When modelling the wireless channel, a popular technique that is commonly used corresponds to the transmission of a *sine tone* at a given frequency, see e.g. [68]. The received power is then modelled as a random variable. The corresponding distribution has been widely studied in the literature from measurements, and the empirical data have shown that the two most common distributions are Rayleigh an Rice [44, 45]. Hence, using similar ideas as in [50], in this example we formulate the channel distribution as a discrete sum based on a Rayleigh and a Rice component to determine the nature of the wireless channel.

First, the discrete mixture that we want to adjust from data is given by

$$\begin{array}{c} p\left(x|\theta \right)=\lambda_{1}p_{\text{Rayleigh}}\left(x|\sigma_{1}^{2}\right)+\lambda_{2}p_{\text{Rice}}\left(x|v,\sigma_{2}^{2}\right), \end{array}$$ (67)

where

$$\begin{array}{cc} p_{\text{Rayleigh}}\left(x|\sigma_{1}^{2}\right) & =\frac{x}{\sigma_{1}^{2}}e^{-\frac{x^{2}}{2\sigma_{1}^{2}}}, \end{array}$$ (68)

$$\begin{array}{cc} p_{\text{Rice}}\left(x|v,\sigma_{2}^{2}\right) & =\frac{x}{\sigma_{”}^{2}}e^{-\frac{\left(x^{2}+v^{2}\right)}{2\sigma_{2}^{2}}}I_{0}(\frac{xv}{\sigma_{2}^{2}}), \end{array}$$ (69)

$$\theta =[\sigma_{1}^{2},\lambda_{1},v,\sigma_{2}^{2},\lambda_{2}],$$ (70)

and *I*_0_(⋅) is the modified Bessel function of zeroth order. In addition, we must also include the constraint λ_1_+ λ_2_ = 1 so *p*(*x*) is a pdf. Thus, we can directly apply our proposed approach by assuming that each measurement point can be associated with a hidden variable that describes if such data point comes from the Rayleigh component or the Rice component, as it is traditionally formulated when dealing with discrete mixtures [46]. Hence, the auxiliary function $Q(\theta ,{\hat{\theta }}^{\left(i\right)})$ is given by

$$\begin{array}{c} Q\left(\theta ,{\hat{\theta }}^{\left(i\right)}\right)=\sum_{t=1}^{2}\sum_{j=1}^{2}\zeta_{tj}^{\left(i\right)}\text{log}\;\lambda_{j}+\sum_{t=1}^{N}\sum_{j=1}^{2}\zeta_{tj}^{\left(i\right)}\text{log}\;f_{j}\left(x_{t},\theta_{j}\right), \end{array}$$ (71)

where ${\hat{\theta }}^{\left(i\right)}$ is the current estimate, *ζ* is the unobserved (*hidden*) data and *ζ*_*tj*_ is an indicator parameter such that *ζ*_*tj*_ = 1 if the *t*-th observation comes from component *j* and is zero otherwise. It is given by:

$$\begin{array}{c} \zeta_{tj}^{\left(i\right)}=\frac{\lambda_{j}^{\left(i\right)}f_{j}\left(x_{t},\theta_{j}^{\left(i\right)}\right)}{\sum_{l=1}^{2}\lambda_{l}^{\left(i\right)}f_{l}\left(x_{t},\theta_{l}^{\left(i\right)}\right)}. \end{array}$$ (72)

The estimate ${\hat{\theta }}^{\left(i+1\right)}$ at the next iteration is then given by:

$$\begin{array}{c} {\hat{\theta }}^{\left(i+1\right)}=\arg\max_{\theta }\;Q\left(\theta ,{\hat{\theta }}^{\left(i\right)}\right), \end{array}$$ (73)

from which we obtain the following expressions:

$${\hat{v}}_{j}^{\left(i+1\right)}=\frac{\sum_{t=1}^{N}\zeta_{tj}^{\left(i\right)}x_{t}\frac{I_{1}(\rho_{tj}^{\left(i\right)})}{I_{0}(\rho_{tj}^{\left(i\right)})}}{P_{j}\left({\hat{\beta }}_{j}^{\left(i\right)}\right)}$$ (74)

$$[{\hat{\sigma }}_{j}^{2}]{}^{\left(i+1\right)}=\frac{\sum_{t=1}^{N}\zeta_{tj}^{\left(i\right)}[x_{t}^{2}+[{\hat{v}}_{j}^{2}]{}^{\left(i\right)}-2x_{t}{\hat{v}}_{j}^{\left(i\right)}\frac{I_{1}(\rho_{tj}^{\left(i\right)})}{I_{0}(\rho_{tj}^{\left(i\right)})}]}{2P_{j}\left({\hat{\beta }}_{j}^{\left(i\right)}\right)}$$ (75)

$${\hat{\lambda }}_{j}^{\left(i+1\right)}=\frac{P_{j}({\hat{\beta }}_{j}^{\left(i\right)})}{\sum_{l=1}^{2}P_{l}({\hat{\beta }}_{l}^{\left(i\right)})}$$ (76)

with

$$\rho_{tj}^{\left(i\right)}=\frac{x_{t}{\hat{v}}_{j}^{\left(i\right)}}{[{\hat{\sigma }}_{j}^{2}]{}^{\left(i\right)}}$$ (77)

$$P_{j}({\hat{\beta }}_{j}^{\left(i\right)})=\sum_{t=1}^{N}\zeta_{tj}^{\left(i\right)}$$ (78)

We also consider the utilization of Akaike’s Information Criterion (AIC) in order to obtain an accurate yet simple model and, thus, discriminating from a Rayleigh channel, a Rice channel, and a mixture of both.

With the above formulation, we consider two cases: a Rayleigh distributed channel and a Rice distributed channel.

#### 6.2.1 Rayleigh distributed channel

**Fig 3:**
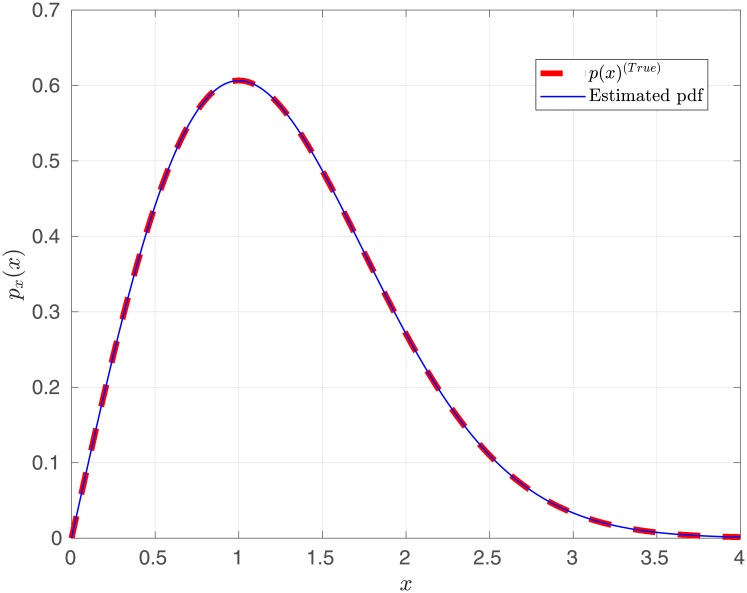
Rayleigh distribution estimation using a Rayleigh-Rice mixture.

In this example, the random variable *x* is drawn from the Rayleigh distribution

$$\begin{array}{c} p{\left(x\right)}^{\left(\text{True}\right)}=\frac{x}{\sigma^{2}}\exp(-\frac{x^{2}}{2\sigma^{2}}) \end{array}$$ (79)

with *σ*^2^ = 1, using the *Slice Sampler* [69]. The best estimated model corresponds to the single Rayleigh component in the mixture. The corresponding estimation of *σ*^2^ yields ${\hat{\sigma }}_{1}^{2}=1.0007\pm 9.003\times 10^{-4}$. Fig 3 shows the *true* Rayleigh distribution and the mean estimated pdf from the 50 MC simulations. We observe an important agreement between the true pdf and the estimated model.

#### 6.2.2 Rice distributed channel

**Fig 4:**
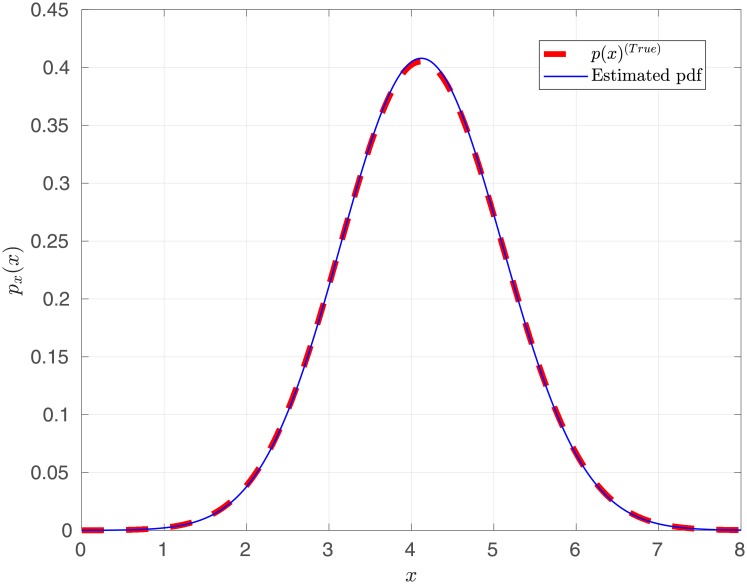
Rice distribution estimation using a Rayleigh-Rice mixture.

In this case, the data is drawn from the Rice distribution

$$\begin{array}{c} p{\left(x\right)}^{\left(\text{True}\right)}=\frac{x}{\sigma^{2}}\exp(-\frac{x^{2}+v^{2}}{2\sigma^{2}})I_{0}(\frac{xv}{\sigma^{2}}) \end{array}$$ (80)

with *v* = 4 and *σ*^2^ = 1, using the *Slice Sampler*. The best model is selected as a single Rician component. The corresponding estimated parameters are ${\hat{v}}_{2}=4.003\pm 1.3\times 10^{-3}$ and ${\hat{\sigma }}_{2}^{2}=0.9858\pm 2.2\times 10^{-3}$. In Fig 4 we show the *true* Rice distribution and the mean estimated pdf from 50 MC simulations. We can observe that the estimator exhibits a good performance for the estimation of a Rice distribution.

## Conclusions and future work

In this paper we have presented a systematic approach for constructing surrogate functions in a wide range of inference problems. Our approach can be utilized for constructing surrogate functions for both the cost function and the constraints, generalizing the popular EM and MM algorithms. Our approach is based on the utilization of data augmentation and kernel functions, yielding simple optimization algorithms when the kernel can be expressed as VMGM. We have shown how our proposal can be utilized to solve inverse problems that are expressed as integral equations and mixture distributions.

In addition, we have shown that our approach can be utilised for constrained/penalized ML and MAP estimations problems. In particular, common problems in statistical inference can directly be solved using our proposal since they can be posed as Variance Mean Gaussian Mixtures (VMGM), yielding quadratic surrogate functions.

In the last two decades the problem of sparse estimation has attracted a lot of attention. Since our approach can be utilized in those problems, and since it is based on the principles of the MM algorithm, a detailed analysis can be done in terms of accuracy and convergence of our technique, and compared against other techniques, such as the ones in [28, 30], and [29], where different Lasso-type problems are compared, the MM algorithm is utilized in constrained problems for ML estimation in generalized linear model regression, and the MM algorithm is used for (unconstrained) sparse estimation under non-convex penalties, respectively.

## Supporting information

S1 Data Set Monte Carlo simulations for Examples 1 and 2. (ZIP)
